# Foliar Application of Salicylic Acid Improves Salt Tolerance of Sorghum (*Sorghum bicolor* (L.) Moench)

**DOI:** 10.3390/plants11030368

**Published:** 2022-01-28

**Authors:** Ahmad Rajabi Dehnavi, Morteza Zahedi, Agnieszka Ludwiczak, Agnieszka Piernik

**Affiliations:** 1 Department of Geobotany and Landscape Planning, Faculty of Biology and Veterinary Sciences, Nicolaus Copernicus University in Torun, 87-100 Torun, Poland; agnieszka.lud@umk.pl; 2 Department of Agronomy and Plant Breeding, College of Agriculture, Isfahan University of Technology, Isfahan 84156-83111, Iran; mzahedi@cc.iut.ac.ir

**Keywords:** sorghum, salinity, exogenous application of salicylic acid, antioxidant enzymes activities, proline

## Abstract

It has been reported that around the world, approximately 19.5% of all irrigated land and 2.1% of dry land is affected by salt stress, and these percentages continue to increase. Sorghum is the fifth most important cereal in the world and therefore research on its salt tolerance is of global importance. In our research, we focused on foliar application of salicylic acid (SA) on salt-stressed sorghum. We performed a pot experiment with two salt levels (0 and 100 mM sodium chloride NaCl) and five SA concentrations (0, 50, 100, 150 and 200 mg/L). Our results suggest that in saline conditions foliar application of SA induced an adaptive response to salinity by inducing proline accumulation as well as antioxidant enzymes activities and enhanced the protection of the photosynthetic machinery, maintained photosynthesis activities, and improved the growth of sorghum plants. These alleviation effects were depended on applied SA concentration. Under saline condition 150 mg/L, SA was the most effective for relieving the adverse effect of salt stress. Under non-saline conditions 100 mg/L SA was the best for improving sorghum growth and dry matter production. Our results demonstrated that foliar SA application is effective in improving sorghum growth under salinity.

## 1. Introduction

Salinity is one of the most important abiotic factors that limit crop growth and final yield mainly in arid and semi-arid regions [1]. It is estimated that around the world about 19.5% of irrigated and 2.1% of dry lands have salinity problems and are continuously increasing mainly because of mishandled irrigation [1,2,3]. Under saline conditions, crops growth and development are inhibited mainly by water stress (physiological drought), ionic toxicity (high accumulation of Na^+^, Cl^−^ in plant tissues), and overproduction of reactive oxygen species (ROS) such as superoxide, hydrogen peroxide, hydroxyl and singlet oxygen in chloroplasts, mitochondria, and apoplectic space. Overall, this stress causes nutrient imbalance, enzymatic and metabolic inhibition and the alteration levels of growth regulators and ultimately reduce plant growth and yield [4,5]. Plants use various defense mechanisms to survive and maintain growth against environmental stress such as salinity. In response to ROS, plants have efficient systems for scavenging free oxygen radicals that protect them from oxidative stress [6,7]. One of the most important protective mechanisms is composed of non-enzymatic antioxidants (glutathione, ascorbate, carotenoids, and tocopherol) and antioxidant enzymes such as catalase (CAT; EC 1.11.1.6.), peroxidase (POD; EC 1.11.1.7), superoxide dismutase (SOD; EC 1.15.1.1) and glutathione reductase (GR; EC 1.6.4.2), which are used to defuse and to scavenge ROS that are accumulated by salt stress induction [8,9]. Indeed, high levels of antioxidant enzymes activities in plant tissues have been shown to provide plant resistance and reduce the damage caused by oxidative stress [9,10]. In addition, plants accumulate compatible soluble compounds, including carbohydrates, amino acids, proline, and proteins to maintain the ionic balance of vacuole, removal of free radicals and the protection of macromolecules and plant organelles [7,11,12]. Proline is a nonessential amino acid of which concentration has increased in many plant species [11,12]. In addition, proline can play a role as a compatible solute, an osmoprotectant, a protective compound of cytosolic enzymes and cellular tissue, a source of nitrogen and carbon, a membrane stabilizer and ROS scavenger [7,13]. This osmotic adjustment regulates the water uptake and cell turgor, which leads to the preservation of various physiological processes, including cell growth and development, and photosynthesis [14,15,16]. Furthermore, in saline conditions maintaining cellular ions homeostasis (especially Na^+^/K^+^) is critical for plant survival. In this case, ion uptake and compartmentalization is another protective mechanism against salinity [17,18]. In this defense mechanism, the accumulation of toxic ions of Na^+^ and Cl^−^ occurs either in the form of transfer to the vacuole or in the form of accumulation in other parts of plant tissue, which causes less damage to vital parts of the plant [17,18]. It is well documented that the salt overly sensitive (SOS) stress signaling pathway has a critical role in ion homeostasis and salt tolerance [17,18]. The SOS regulatory pathway affects ions transporters and thus regulates ions Na^+^ and K^+^ homeostasis [18].

Although breeding for salt tolerance is important, because of the lack of reliable traits for selection and the multi-genetic source of salinity tolerance, there has been no significant success. It seems that using other methods that are cost-time-effective are more suitable to reduce the harmful effects of salinity and should be taken into consideration [19]. Various studies have shown that exogenous application of growth regulators can reduce the harmful effects of salinity on plants, and it seems to be a practical way to cope with salt stress [20,21,22]. Salicylic acid (SA) is a plant phenolic compound that stimulates various biological processes in plants as a growth regulator [23,24]. It can regulate germination [25,26], absorption and transfer of ions [26,27], membrane permeability [28,29], stomatal conductance and transpiration and photosynthetic activities [30,31]. In addition, SA plays an important role in defense mechanisms against environmental stresses including salinity [32,33]. Exogenous application of SA extensively has been reported to alleviate the harmful effects of salinity [21,33,34]. For example, Noreen [34] states that SA is the most effective phytoprotectant compound that reduces the effects of salt-induced osmotic and ionic stress and causes salt tolerance in many plant species. However, the extent of these ameliorative effects depends on the stage of plant development and the applied SA concentration, which is poorly investigated. 

Sorghum (*Sorghum bicolor* L.) is one of the five most important cereals in the world and has the potential to be used in stress areas [35]. Therefore, research on its salt tolerance is of global importance. To breed plants such as sorghum for salt tolerance, study on the metabolic changes in saline conditions to introduce physiological, biochemical, and molecular markers is necessary [36,37]. In addition, although the positive effect of SA on reducing adverse effects of salinity on the sorghum plant has been already reported, mechanisms involved in alleviating the effect of SA and the effect of applied SA concentrations and optimal SA concentration have not been well identified. Therefore, our aims are to investigate responses of the sorghum plants to salinity and the role of different applied SA concentrations in improving tolerance of this plant against salt stress. We assumed that: (1) SA have a positive effect on sorghum salt tolerance (2) different levels of SA have a different effect, (3) it is possible to select the best SA concentration for future agricultural purpose.

## 2. Results

### 2.1. Ionic Balance

**Figure 1:**
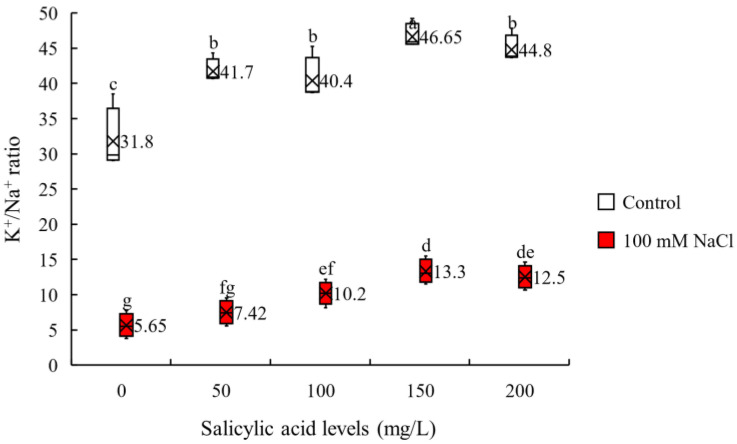
K^+^/Na^+^ ratio of sorghum leaves for the interaction of SA treatments × salinity (100 mM NaCl) in four repetitions at milky seeds stage. Different letters indicate significant differences by HSD at *p* < 0.05. The data for analysis are given in Table S1.

To investigate the effect of salinity and SA application on the content of two critical sorghum elements and their ratio, we measure K^+^ and Na^+^ content and calculate K^+^/Na^+^ ratio. It is generally accepted that maintaining high K^+^/Na^+^ is a necessity for salinity tolerance as this guarantees optimal cellular metabolic functions. We found that the interaction effects of salinity and SA application were significant on shoot K^+^/Na^+^ ratio (Table 1). We observed that K^+^/Na^+^ ratio was decreased by salinity under all SA concentrations. However, SA application increased K^+^/Na^+^ ratio under both non-saline and saline conditions. Our results demonstrate that the highest increases in K^+^/Na^+^ ratio under both non-saline (46%) and saline (135%) conditions is achieved at 150 mg/L SA (Figure 1 and Figure S1). Moreover, the maximum K^+^/Na^+^ (46.5) was obtained at 150 mg/L SA under control treatment and the minimum value (5.65) was achieved under saline condition in the non-sprayed plants (Figure 1 and Table S1). These results show that SA can positively affect K^+^/Na^+^ ratio under saline condition.

### 2.2. Photosynthetic Parameters

**Figure 2:**
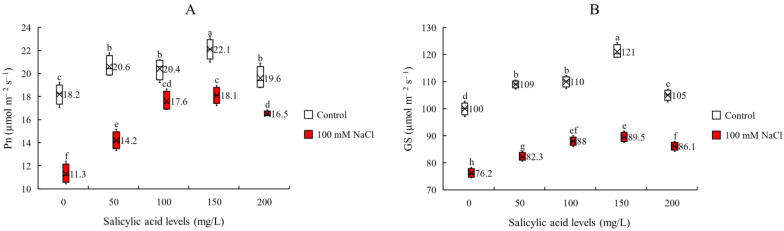
(**A**) photosynthetic rate (Pn) and (**B**) stomatal conductance (Gs) of sorghum plants for the interaction of SA treatments × salinity (100 mM NaCl) in four repetitions at fifty percent flowering stage. Different letters indicate significant differences by HSD at *p* < 0.05. The data for analysis are given in Table S1.

Photosynthetic performance is determined by different parameters such as photosynthetic rate (Pn), stomatal conductance (Gs) and intercellular CO_2_ concentration (Ci). Thus, we monitored the effect of salinity and foliar application of SA on these parameters to assess how they are affected. We identified that the interaction effects of salinity and SA application were significant on Pn and Gs but non-significant on Ci (Table 1). Here we found that although salinity reduced Pn and Gs under all SA treatments, but SA application ameliorated this negative effect. However, there were significant differences between SA-applied concentrations (Figure 2). In both non-saline and saline conditions, the most effective SA concentrations for enhancing Pn and Gs were 150 mg/L (Figure 2A,B, Table S1). According to the results, it seems that SA, by affecting photosynthetic stomatal factors and photosynthetic rate, enhanced photosynthetic performance under salt stress condition. However, this positive effect depends on the applied concentration.

### 2.3. Photosynthetic Pigments

**Figure 3:**
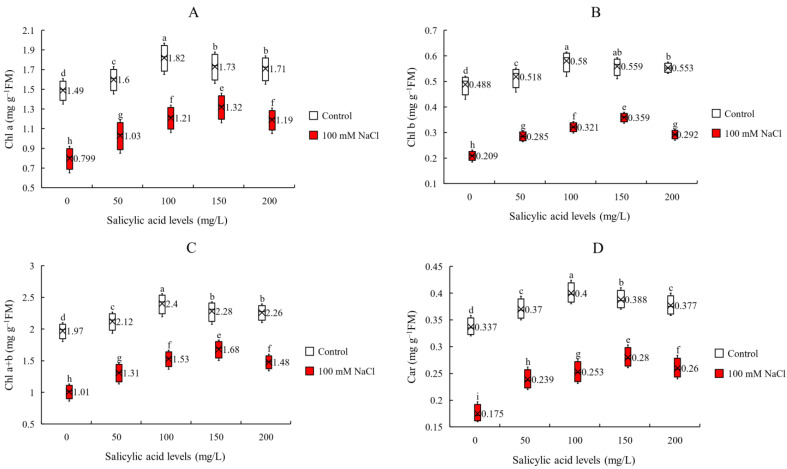
The contents of (**A**) chlorophyll a (Chl a), (**B**) chlorophyll b (Chl b), (**C**) total chlorophyll (Chl a + b) and (**D**) carotenoids (Car) of sorghum plants for the interaction of SA treatments × salinity (100 mM NaCl) in four repetitions at fifty percent flowering stage. Different letters indicate significant differences by HSD at *p* < 0.05. The data for analysis are given in Table S1.

Photosynthetic pigments are other factors that can affect photosynthetic performance, plants growth and development. To investigate the effect of salt stress and exogenous application of SA on photosynthetic pigments in sorghum plants we measured Chl a, Chl b, Chl a + b, and Car contents. We found that the interaction effects of salinity and SA foliar application were significant on Chl a, Chl b, Chl a + b and Car (Table 1). We observed that salinity negatively affected photosynthetic pigments. However, compared to the control foliar application of SA improved Chl a, Chl b, Chl a + b and Car content under both none-saline and saline conditions which was dependedant on SA applied concentrations. Under saline conditions, the most effective SA concentration was 150 mg/L which causes the highest increases in Chl a, Chl b, Chl a + b and Car (65 and 72, 66 and 60% respectively) (Figure 3A–D). However, under non-saline the highest increases in Chl a, Chl b, Chl a + b and Car (22, 19, 22 and 19%, respectively) were achieved at 100 mg/L SA (Figure 3 and Table S1). Under saline treatment, the maximum values of Chl a, Chl b, Chl a + b and Car (1.32, 0.359, 1.68 and 0.280 mg g^−1^ FM respectively) were noted at 150 mg/L SA (Figure 3 and Table S1). This suggests that SA by preserving photoreceptors from the negative effect of salt stress maintains the photosynthetic capacity and performance and 150 150 mg/L SA is the best dose. 

### 2.4. Antioxidant Enzymes Activities

**Figure 4:**
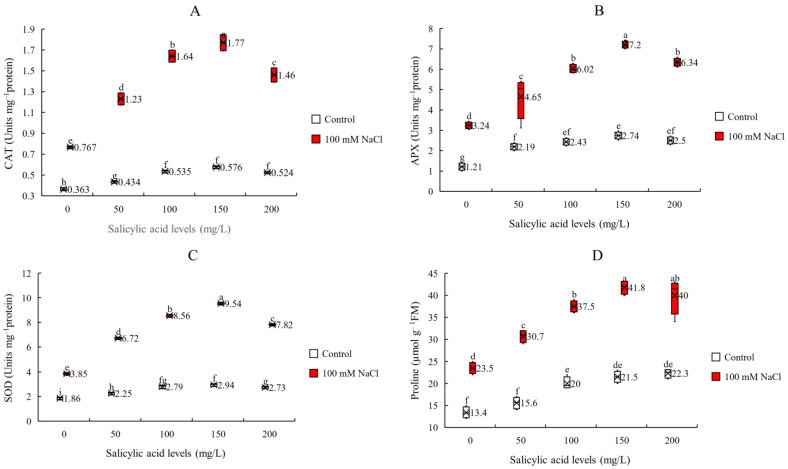
Specific activity of antioxidative enzymes (**A**) catalase (CAT), (**B**) ascorbate peroxidase (APX), and (**C**) superoxide dismutase (SOD) and (**D**) proline content (P) of sorghum plants for interaction of SA treatments × salinity (100 mM NaCl) in four repetitions at fifty percent flowering stage. Different letters indicate significant differences by HSD at *p* < 0.05. The data for analysis are given in Table S1.

Antioxidant enzymes activities are one of the most important defense mechanisms against stress such as salinity. So, evaluating the response of these enzymes to salinity and SA can help us in better understanding SA induced adaptive response. Thus, we measured CAT, APX and SOD activities. Our results show that the interaction effects of salinity and foliar application of SA significantly enhanced these enzymes activities (Figure 4, Table 1). However, the enhancing effect was more noticeable in the saline condition, but it was dependedant on SA applied concentrations. We observed that under saline condition, 150 mg/L SA causes the highest increase in all three enzymes activities. The corresponding values in increasing activities for CAT, APX and SOD were 148, 130 and 122% respectively (Figure 4 and Table S1). The lowest defense mechanisms against salinity were observed under salinity without SA application (with minimum values of CAT, APX and SOD −0.767, 3.24 and 3.85 unit mg^−1^ protein respectively). These results suggest that SA by improving the activity of antioxidant enzymes helps plants to cope with salt stress more effectively and the most effective concentration is 150 mg/L SA.

### 2.5. Osmolyte

Osmolyte accumulation is another mechanism against osmotic stress during salinity. To investigate the effect of salinity and SA on osmoporotectant accumulation, we measured proline content as an osmolyte marker. Our results show that the interaction effects of salinity and SA were significant (Table 1). Here we found that foliar application of SA increased proline content under both non-saline and saline conditions. The highest increase in proline content under non-saline condition (66%) was achieved at 200 mg/L SA, but under saline condition (78%) was obtained at 150 mg/L SA (Figure 4D and Table S1). Under saline condition, the maximum value of proline content (41.8 µmol g^−1^ FM) was obtained at 150 mg/L SA and the minimum value (23.5 µmol g^−1^ FM) was achieved in non-sprayed plants (Figure 4D and Table S1). According to the results, it seems that SA reduces the negative effect of osmotic stress of salinity by enhancing proline accumulation to maintain turgor pressure inside the cells. We found the most effective SA concentration of 150 mg/L.

### 2.6. Growth Parameters

**Figure 5:**
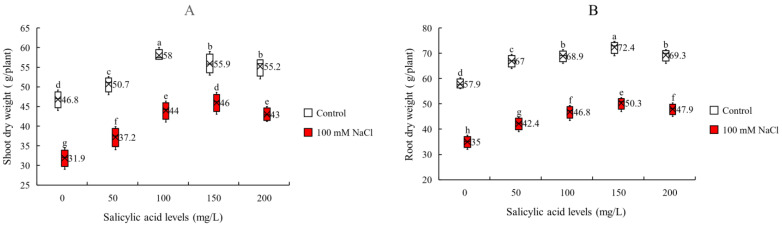
(**A**) shoot dry weight (SDW) and (**B**) root dry weight (RDW) of sorghum plants for interaction of SA treatments × salinity (100 mM NaCl) in four repetitions at fifty percent flowering stage. Different letters indicate significant differences by HSD at *p* < 0.05. The data for analysis are given in Table S1.

The outcome of all modulatory effects of SA in plants under salinity can be traced by evaluating its growth and biomass accumulation. To achieve this purpose, we measured shoot dry weight (SDW) and root dry weight (RDW). We found that the interaction effects of salinity and SA foliar application were significant (Table 1). We observed that salinity reduced SDW and RDW. However, foliar application of SA significantly improved SDW and RDW not only in saline, but also in non-saline conditions. Although this positive effect in the non-saline condition was lower than saline condition, it demonstrates that foliar application of SA can enhance plants growth also in normal conditions. Nevertheless, this effect was dependent on applied SA concentration and the highest increases in SDW and RDW were achieved at 100 and 150 mg/L SA (24 and 25% respectively). In saline condition, the highest increases (ca. 44%) and the maximum values for SDW and RDW (46.0 and 50.3 g/plant respectively) were achieved at 150 mg/L SA (Figure 5 and Table S1).

## 3. Discussion

This study was conducted to investigate responses of the sorghum plants to salinity and the role of different applied SA concentrations in improving its tolerance to salt stress. In general, we observed that salinity caused significant changes in the physiological and biochemical attributes leading to ultimately reduced shoot and root biomass production. These reductions were to some extent greater in roots. In line with our results, various experiments have reported reductions in plant growth parameters under saline conditions [38,39]. For example, Asghar Dashti et al. [40], Kafi et al. [41] and Rajabi Dehnavi et al. [42] have reported that salinity significantly reduced root and shoot growth of sorghum plants. Our results demonstrate that under saline condition, biomass production had the highest correlation with Pn (r = 0.995) (Table 2), which indicates that under saline condition, any change in photosynthetic performance strongly affects dry matter accumulation. Photosynthesis is an important metabolic pathway that regulates plant growth and is approved as one important target of salinity [43,44]. It is well known that photosynthesis capacity depends on stomatal and non-stomatal factors which can be affected by salinity [5]. In general, salinity by damage to the photosynthetic machinery through various parameters including stomatal operations and gas exchange, pigments, chloroplast development, membrane structure and activity, electron transport and enzyme activities ultimately impede crop production [44,45]. The negative effects of salinity on photosynthesis also can be due to photo inhibition of photosystem II, conformational changes of membrane-bound ATPase enzyme complex [44,46], negative feedback of sink activity [44,47] and disruption of the enzymatic process of CO_2_ fixation [44,48]. Our results show that salinity negatively affects photosynthetic performance by significantly reducing photosynthetic parameters and pigment content. These results are in line with other research in many other plants and sorghum [49,50]. Our results demonstrate that photosynthesis parameter (Pn) was significantly correlated to Gs (r = 0.985) (Table 2). This correlation identifies that under saline condition, limitation in stomatal factors can strongly affect photosynthesis performance. Our results show that in saline condition, Gs was significantly reduced which causes reduction in photosynthesis rate. It is reported that under saline condition, the reduction of cells’ water potential via inducing stomatal closure limits the stomatal and mesophyll diffusion which in turn it reduces CO_2_ pressure and Gs and Pn [47,51]. In addition, Munns and Tester [52] also stated that under saline condition, decreasing Gs in leaves might be due to biosynthesis of ABA in stomatal guard cells. In addition, our results show that K^+^/Na^+^ ratio plays a significant role in maintaining the photosynthetic rate and stomatal performance and this finding confirms previous research [53,54]. Moreover, in our experiment the correlation coefficients between traits show that photosynthesis parameter (Pn) was significantly correlated to Chl a (r = 0.989), Chl b (r = 0.953), and Chl a + b (r = 0.987) (Table 2). Salt stress reduces the contents of Chl a, Chl b, Chl a + b, and Car which causes a reduction in photosynthesis. In line with our results are other findings that salt stress reduces the chlorophyll content and inhibits chlorophyll biosynthesis in sorghum [55,56]. This happens by the inactivation of enzymes involved in the synthesis of photosynthetic pigments [57,58]. Besides, this reduction can be due to ROS generation and increasing chlorophyllase enzyme activity [58,59]. In general, our results demonstrate that salinity by reducing photosynthetic pigments content, disrupting stomatal performance and gas exchange, and changing K^+^/Na^+^ ratio significantly reduces sorghum photosynthesis capacity, growth, and biomass production.

Our results show that foliar application of SA improved shoot and root growth under both none-saline and saline conditions, which is in line with previous research on sorghum [60,61]. However, we demonstrated that the positive effects were greater under saline condition. Based on a high correlation of SDW and RDW with Pn (r = 0.995 and r = 0.975 respectively), it seems that in saline conditions, the stimulatory effect of SA on shoot and root growth is probably due to enhancement in net photosynthetic rate and allocation of more assimilates to them. As previously mentioned, in saline conditions photosynthesis rate was highly correlated with stomatal parameters, photosynthetic pigments content and K^+^/Na^+^ ratio (Table 2). Thus, foliar application of SA by increasing Gs, Ci values and by increasing Chl a, Chl b, and Chl a + b contents moderated the negative effect of salinity on photosynthetic capacity. In line with our results, it has been reported that exogenous application of SA under saline condition improves photosynthetic capacity by regulation of stomatal conductance, and protects and enriches the performance and photosynthetic pigments content [34]. In addition, SA can reduce the adverse effects of salinity stress on photosynthetic capacity by improving important metabolic factors in carbon uptake and fixation such as the concentration and the activity of rubisco, the activity of nitrate reductase and ATP-sulfurylase and nitrate mobilization in tissues [62,63]. 

Furthermore, our results also demonstrated that foliar application of SA increased the proline content of sorghum plants under non-saline and saline conditions. This effect also has been proved in various plants such as wheat [64] and barley [65]. Increasing proline content is known as an indicator to reduce the harmful effects of salinity [66,67,68]. It has been reported that in saline condition, exogenous application of SA increases the activity of proline synthesis enzymes such as pyrroline carboxylic acid and glutamyl kinase and finally increases proline content in cells [69]. El-Tayeb [65] also reported that the enhancement of proline content in SA-treated plants might be owing to dissolved proteins reduction. A high positive correlation coefficient of proline with Pn (r = 0.970) and Gs (r = 0.930) (Table 2) suggested that under saline condition accumulation of proline regulates cell osmotic potential and stomatal conductance and moderates salt effects on stomatal factors which are related to the osmoprotective role of this compound. In addition, high positive correlation coefficient of proline with Chl a (r = 0.982), Chl b (r = 0.906) and Chl a + b (r = 0.971) (Table 2) show that proline accumulation can preserve pigments from negative effects of salinity. These effects are related to the proline role as a compound in the non-enzymatic antioxidant defense system for inhibiting singlet oxygen formation [70]. Furthermore, during salt stress proline also can decrease toxic ion absorption by reduction of cell osmotic potential [71,72] which can be confirmed by the high positive correlation coefficient between proline accumulation and K^+^/Na^+^ ratio (r = 0.977) (Table 2).

We proved that foliar application of SA enhanced anti-oxidative defense mechanisms of sorghum against salt stress, i.e., CAT, APX and SOD activities under non-saline and saline conditions. However, the effect of SA on CAT and SOD activities were more significant under saline condition, when the effect on APX was more significant under none-saline condition. In fact, it has been reported that throughout acclimation to salt stress, exogenous application of SA increases the accumulation of ROS (especially H_2_O_2_) in plants [73]. Generation of H_2_O_2_ by SA induced the anti-oxidative defense mechanisms including enzymatic and non-enzymatic antioxidants [74]. This induction by improving plants’ metabolic activity improves plants’ tolerance to salinity [75]. We observed that under saline condition the correlations of all three antioxidant enzymes activities with photosynthetic parameters, photosynthetic pigments, and growth parameters were high (Table 2). Thus, it can be suggested that in addition to induction of proline accumulation, the raised level of antioxidant enzymes activities due to SA application also might be responsible for increasing tolerance of sorghum plants to NaCl stress.

Nevertheless, the positive effects of SA on modulating effects of salinity depend on its applied concentration. We observed that with increasing SA levels from 0 to 200 mg/L K^+^/Na^+^ ratio, Pn, Gs and Ci, Chl a, Chl b, Chl a + b and Car content, proline content, CAT, APX and SOD activities and SDW and RDW are enhanced under both none-saline and saline conditions. However, the effective concentration under non-saline condition was 100 mg/L SA and under saline condition was 150 mg/L SA.

## 4. Materials and Methods

A pot experiment as a complete randomized design in 2 × 5 factorial, two salt levels (control group and 100 mM NaCl) and five concentrations of SA (0, 50, 100, 150 and 200 mg/L) with 4 replications was undertaken in an open growth chamber (under non controlled condition) in a field at Faculty of Agriculture, Isfahan University of Technology (IUT) (32°32′ N, 51°23′ E, 1630 m above mean sea level, 14.5 °C mean annual temperature, and 140 mm mean annual precipitation), Isfahan, Iran during the spring and summer of 2021. During this experiment, the average daily temperature was between 25 and 34 °C, and at night it was about 20 to 23 °C. The region is under dry and semi-dry climate conditions, with high temperatures and low rainfall during the spring and summer (Table 3).

Sorghum seeds (10 seeds of Speed Feed cultivar which obtained from Seed and Plant Improvement Institute (SPII) Karaj, Alborz Province, Iran) were planted in plastic pots (25 cm diameter × 20 cm depth) that contained clay-loam soil (35% clay, 35% silt and 30% sand) and after full emergence, the number of plants was reduced to 4 seedlings per pot and salinity treatment commence. To prevent salt shock to plants, saline treatment was stepped up. About 500 mL of solution was given to each pot at each irrigation time (three times per week). Four weeks after subjecting the plants to salinity levels, the SA was sprayed on the foliage at two steps, 7 days apart. At two weeks after second foliar application (approximately at fifty percent flowering stage) chlorophyll content, proline content, photosynthesis parameters, and antioxidant enzymes activities were measured in the leaves by the following methods:

### 4.1. Photosynthesis Measurement

Photosynthetic rate (Pn), stomatal conductance (Gs) and intercellular CO_2_ concentration (Ci) were determined by using a gas analyzer portable photosynthetic system (LI-COR 6400, LI-COR, and Lincoln, Lincoln, NE, USA) from 9 to 11 a.m.

### 4.2. Photosynthetic Pigments Content

Photosynthetic pigments content was measured by the method of Lichtenthaler and Buschmann [76]. In this method, 0.5 g of the fresh leaf was mixed with 10 mL of 80% acetone. The extracted sample was filtered using Whatman filter paper. The homogenate was centrifuged at 5000 rpm for 15 min. Then the contents of each test tube were made to 10 mL with 80% acetone and then the amount of light absorption of each extract was read by spectrophotometer at wavelength 663, 646 and 470 nm. 80% acetone (without plant extract) was used to blank the spectrophotometer. Finally, the contents of Chlorophyll chlorophyll a (Chl a), chlorophyll and b (Chl a, (Chl b), and total chlorophyll (Chl a + b) and carotenoids (Car) in leaves as mg g^−1^ FW were calculated using the following equations:

$$Cholorophyll\left(a\right)=\frac{\left[\left(12.21\times Abs663\right)-\left(2.81\times Abs646\right)\times mlAseton\right]}{mgLeaf}$$

$$Cholorophyll\left(b\right)=\frac{\left[\left(20.13\times Abs646\right)-\left(5.03\times Abs663\right)\times mlAseton\right]}{mgLeaf}$$

$$Carotenoids=\frac{\left(\left[\left(1000\times Abs470-3.27\left(Chla\right)-104\left(Chlb\right)\right)/227\right]\times mlAseton\right)}{mgLeaf}$$

### 4.3. Measurement of Antioxidant Enzymes Activities

To measure the specific activity of antioxidant enzymes, first, 100 mg of fresh sample was mixed with 1 mL extraction buffer (which consists of polyvinyl pyrrolidone 1%, Triton X100 0.05% and potassium phosphate buffer 100 mM (7 = pH)) and homogenized completely. The homogenate was centrifuged at a rate of 15,000 rpm at 4 °C for 20 min. The clear part at the top of the extract was used to assay the specific activity of antioxidant enzymes.

CAT activity was measured by following the reduction of H_2_O_2_ at 240 nm according to the method of Alici and Arabaci [77]. In this method, 2.95 mL of reaction buffer, including 50 mM potassium phosphate buffer (pH = 7) and 15 mM hydrogen peroxide, were mixed with 0.05 mL of enzyme extract. The specific activity of catalase enzyme was calculated by dividing the volumetric activity of catalase by the protein content in the extract which was determined by the Bradford (1976) method [78]. APX activity was determined spectrophotometrically by monitoring the decrease in ascorbate at 290 nm by using the method of Nakano and Asada [79]. To start the reaction, 2.95 mL of reaction buffer including phosphate potassium buffer 50 mM (pH = 7), hydrogen peroxide 0.5 mM, ascorbate 5 mM was mixed with 0.05 mL of enzyme extract. The APX specific activity was calculated according to the method described in the CAT enzyme assay. SOD activity was measured spectrophotometrically based on inhibition in the photochemical reduction of nitroblue tetrazolium by using the Giannopolitis and Ries method [80]. To prepare the reaction solution, 50 μL of the enzyme extract was added to 3 mL of the reaction buffer, including phosphate buffer 50 mM (pH = 7.8), Ethylenediaminetetraacetic acid 75 nM, methionine 13 mM, nitroblobotrazolium 63 μL and riboflavin 1.3 μM. The reaction solution was placed for 15 min under two 15-watt fluorescent lamps with a height of 20 cm with a light intensity of 1000 lux, and the reaction was terminated by turning off the lamps. After that absorption at wavelength 560 nm is measured. The SOD-specific activity was calculated according to the method described in the CAT enzyme assay. 

### 4.4. Proline Content

Proline level was measured according to the Bates et al. method [81] based on proline reaction with ninhydrin. 0.5 g fresh leaf samples from each treatment were homogenized in 0.5 mL of 3% (*w*/*v*) sulphosalicylic acid. After that 0.2 mL of each homogenate was mixed with 0.2 mL of glacial acetic acid to which 0.2 mL of ninhydrin was added. The reaction mixture was boiled in a water bath at 100 °C for 30 min and immediately cooled in an ice bath. After cooling, 0.4 mL of toluene was added to the reaction mixture. After thorough mixing, the chromophore containing toluene was separated and absorbance of red color developed was read at 520 nm against toluene.

### 4.5. Shoot and Root Dry Weight

At four weeks after the second foliar application (approximately at milky seeds stage) shoot dry weight (SDW) and root dry weight (RDW) were determined from the harvested portion in each after the samples were oven-dried for 72 h at 72 °C and expressed as g/plant.

### 4.6. K^+^/Na^+^ Ratio

For K^+^/Na^+^ ratio, at the end of the experiment, the dried samples were grounded. One gram of each ground sample was weighed and placed in an electric oven at 550 °C for 6 h until the organic matter was completely burned and changed to ashes. Ashe material was digested in an acid mixture consisting of sulfuric, nitric and per chloric acids in the ratio of 1:8:1 (*v*/*v*). The aliquot was filtered and used for the determination of potassium and sodium ions with a flame photometer [82].

### 4.7. Statistical Analysis

Statistical Analysis Software version 9.4 (SAS Institute Inc., Cary, NC, USA) was used. For detecting differences among the treatments and statistically significant effects, we used two- and one-way ANOVA with Honest significant difference (HSD, *p* ≤ 0.05).

## 5. Conclusions

Our results demonstrated that in saline conditions foliar application of SA induced an adaptive response to salinity and alleviated salt toxicity. In fact, SA induced proline accumulation and enhanced activity of antioxidant enzymes, the protection of the photosynthetic machinery, and then maintained photosynthesis activities and improved the growth of sorghum plants. The complexity of the plant response we proved by correlation results. The alleviation of SA effects was dependedant on applied SA concentration. Under saline condition 150 mg/L was the most effective to relieve the adverse effect of salt stress in sorghum plants. We also observed that in non-saline conditions foliar application of SA improved sorghum growth, development, and dry matter production and 100 mg/L was the best concentration. We believe that it is possible to reduce the harmful effects of salinity by adjusting photosynthetic capacity in sorghum plants by SA. However, because the effect of exogenous application of SA is related to the plant developmental stage, we recommend studying the SA effects in different development stages to better understand these alleviation effects. It also seems that the responses of salt tolerant and sensitive genotypes to foliar application of SA is worth being investigated. It would help our better understanding of SA effects on salt tolerance. We believe that our findings will be useful for future agricultural applications.

## Figures and Tables

**Table 1:** Analysis of variances ANOVA (mean squares) for different parameters of sorghum in two levels salinity and five levels of foliar application salicylic acid in four repetitions.

Trait	Abbreviation	Sources of Variations			
		S	SA	S × SA	Error
	df	1	4	4	30
Ionic balance	K^+^/Na^+^	9768 *	152 *	20.3 *	4.12
Photosynthetic	Pn	212 *	131 *	7.41 *	0.686
parameters	Ci	397 *	116 *	1.12 ^n.s^	2.55
	Gs	6184 *	316 *	49.9 *	4.10
Photosynthetic	Chl a	3.13 *	0.217 *	0.011 *	0.002
pigments	Chl b	0.606 *	0.016 *	0.001 *	0.003
	Chl a + b	6.49 *	0.353 *	0.018 *	0.003
	Car	0.194 *	0.005 *	0.003 *	0.001
Antioxidant enzymes	CAT	7.86 *	0.462 *	0.193 *	0.002
	APX	107 *	9.08 *	2.00 *	0.106
	SOD	229 *	13.8 *	6.14 *	0.012
Osmolytes	P	2528 *	259 *	28.1 *	3.17
Growth	SDW	1669 *	211 *	3.92 *	0.511
parameters	RDW	5111 *	264 *	3.10 *	0.631

K^+^/Na^+^ = K^+^ to Na^+^ ratios in shoot, Pn = photosynthetic rate; Ci = intercellular CO_2_ concentration; Gs = stomatal conductance; Chl a = chlorophyll a; Chl b = chlorophyll b; Chl a + b = total chlorophyll; Car = carotenoids; CAT = catalase; APX = ascorbate peroxidise; SOD = superoxide dismutase; P = proline; SDW = shoot dry weight; RDW = root dry weight; S = salinity; SA = foliar application salicylic acid; df = degrees of freedom; ^n.s^ = non-significant; Error = within group variance; * = *p* ≤ 0.05.

**Table 2:** Correlation coefficients between treats for different parameters of sorghum under non-saline) the bottom lines of the table (and saline conditions (the top lines of the table) in all foliar-applied SA concentrations (for each parameter *n* = 4).

Trait	SDW	RDW	K^+^/Na^+^	Chl a	Chl b	Chl a + b	P	CAT	APX	SOD	Pn	Gs
SDW	1	0.984 *	0.940 *	0.993 *	0.943 *	0.988 *	0.983 *	0.987 *	0.976 *	0.981 *	0.995 *	0.969 *
RDW	0.945 *	1	0.951 *	0.993 *	0.943 *	0.987 *	0.992 *	0.973 *	0.990 *	0.980 *	0.975 *	0.940 *
K^+^/Na^+^	0.871 ^n.s^	0.964 *	1	0.936 *	0.840 ^n.s^	0.922 *	0.977 *	0.890 *	0.975 *	0.891 *	0.905 *	0.840 ^n.s^
Chl a	0.994 *	0.947 *	0.875 ^n.s^	1	0.971 *	0.998 *	0.982 *	0.991 *	0.990 *	0.993 *	0.989 *	0.955 *
Chl b	0.998 *	0.942 *	0.866 ^n.s^	0.998 *	1	0.979 *	0.906 *	0.978 *	0.935 *	0.985 *	0.953 *	0.919 *
Chl a + b	0.994 *	0.952 *	0.885 *	0.999 *	0.997 *	1	0.971 *	0.994 *	0.984 *	0.996 *	0.987 *	0.952 *
P	0.941 *	0.870 ^n.s^	0.847 ^n.s^	0.920 *	0.932 *	0.920 *	1	0.954 *	0.993 *	0.961 *	0.970 *	0.930 *
CAT	0.993 *	0.941 *	0.866 ^n.s^	0.994 *	0.998 *	0.993 *	0.946 *	1	0.970 *	0.997 *	0.994 *	0.975 *
APX	0.951 *	0.998 *	0.963 *	0.942 *	0.941 *	0.950 *	0.887 *	0.943 *	1	0.970 *	0.970 *	0.921 *
SOD	0.993 *	0.942 *	0.864 ^n.s^	0.986 *	0.994 *	0.986 *	0.952 *	0.998 *	0.950 *	1	0.987 *	0.963 *
Pn	0.785 ^n.s^	0.874 ^n.s^	0.820 ^n.s^	0.830 ^n.s^	0.802 ^n.s^	0.835 ^n.s^	0.576 ^n.s^	0.780 ^n.s^	0.844 ^n.s^	0.761 ^n.s^	1	0.985 *
Gs	0.620 ^n.s^	0.590 ^n.s^	0.371 ^n.s^	0.590 ^n.s^	0.603 ^n.s^	0.581 ^n.s^	0.430 ^n.s^	0.610 ^n.s^	0.595 ^n.s^	0.630 ^n.s^	0.555 ^n.s^	1

SDW = shoot dry weight; RDW = root dry weight; K^+^/Na^+^ = K^+^ to Na^+^ ratios in shoot; Chl a = chlorophyll a; Chl b = chlorophyll b; Chl a + b = total chlorophyll; Car = carotenoids; CAT = catalase; APX = ascorbate peroxidise; SOD = superoxide dismutase; Pn = photosynthetic rate; Gs = stomatal conductance; P = proline; ^n.s^ = non-significant; Error = within group variance; * = *p* ≤ 0.05.

**Table 3:** Weather data of the location during experiment period.

Month	Mean Temperature (°C)			Total Precipitation (mm)	Mean Relative Humidity (%)
	Lowest	highest	Average		
May	11	29	20	16	31
June	17	35	26	6	23
July	20	38	29	3	22
August	18	37	27.5	0	24

## Data Availability

The data presented in this study are available on request from the corresponding authors. The data are not publicly available due to ongoing research.

## Supplementary Materials

The following are available online at https://www.mdpi.com/article/10.3390/plants11030368/s1, Table S1: Mean of interaction effects of salinity × foliar application Salicylic acid levels for different parameters of sorghum under two levels of salinity and foliar-applied salicylic acid.
