# Supplementary material for: Foliar Application of Salicylic Acid Improves Salt Tolerance of Sorghum (Sorghum bicolor (L.) Moench)
Source: Plants (Basel). 2022 Jan 28;11(3):368. doi: 10.3390/plants11030368 (PMC8839348; doi:10.3390/plants11030368)
Supplement: Supplementary file 1 [file plants-11-00368-s001.zip › plants-1503671-supplementary.pdf]

Supplementary table S1. Mean of interaction effects of Salinity × foliar application Salicylic acid levels for different parameters of sorghum under two levels of salinity and five foliar-applied Salicylic acid in four repetitions.

STRESS	SA (mg/L)	SDW (gr/plant)	RDW (g/plant)	K^+/Na^+	Chl a (mg g^{-1}FM)	Chl b	Chl a+b	Car	Proline (µmol g^{-1}FM)	CAT (Unit mg^{-1}protein)	APX	SOD	Pn (µmol m^{-2} s^{-1})	Gs (µmol m^{-2} s^{-1})
Control	SA1(0)	46.8 d	57.9 d	31.8 c	1.49 d	0.488 d	1.97 d	0.337d	13.4 f	0.363 h	1.21 g	1.86 i	18.2 c	100 d
	SA2(50)	50.7 c	67.0 c	41.7 b	1.60 c	0.518 c	2.12 c	0.370c	15.6 f	0.434 g	2.19 f	2.25 h	20.6 b	109 b
		(+8)	(+16)	(+31)	(+7)	(+6)	(+7)	(+10)	(+16)	(+19)	(+126)	(+21)	(+13)	(+9)
	SA3(100)	58.0 a	68.9 b	40.4 b	1.82 a	0.580 a	2.40 a	0.400a	20.0 e	0.535 f	2.43 ef	2.79 fg	20.4 b	110 b
		(+24)	(+19)	(+27)	(+22)	(+19)	(+22)	(+19)	(+49)	(+47)	(+127)	(+50)	(+12)	(+10)
	SA4(150)	55.9 b	72.4 a	46.65 a	1.73 b	0.559 ab	2.28 b	0.388b	21.5 de	0.576 f	2.74 e	2.94 f	22.1 a	121 a
		(+19)	(+25)	(+46)	(+16)	(+14)	(+16)	(+15)	(+60)	(+58)	(+100)	(+58)	(+21)	(+21)
	SA5(200)	55.2 b	69.3 b	44.8 a	1.71 b	0.553 b	2.26 b	0.377c	22.3 de	0.524 f	2.50 ef	2.73 g	19.6 b	105 c
		(+18)	(20+)	(+41)	(+15)	(+13)	(+15)	(+12)	(+66)	(+44)	(+81)	(+47)	(+7)	(+5)
Salinity	SA1(0)	31.9 g	35.0 h	5.65 g	0.799 h	0.209 h	1.01 h	0.175i	23.5 d	0.767 e	3.24 d	3.85 e	11.3 f	76.2 h
	SA2(50)	37.2 f	42.4 g	7.42 fg	1.03g	0.285 g	1.31 g	0.239h	30.7 c	1.23 d	4.65 c	6.72 d	14.2 e	82.3 g
		(+17)	(+12)	(+31)	(+29)	(+36)	(+30)	(+36)	(+31)	(+60)	(+85)	(+74)	(+25)	(+8)
	SA3(100)	44.0 e	46.8 f	10.2 ef	1.21 f	0.321 f	1.53 f	0.253g	37.5 b	1.64 b	6.02 b	8.56 b	17.6 cd	88 ef
		(+38)	(+34)	(+80)	(+51)	(+53)	(+51)	(+44)	(+59)	(+114)	(+86)	(+122)	(+56)	(+15)
	SA4(150)	46.0 d	50.3 e	13.3 d	1.32 e	0.359 e	1.68 e	0.280e	41.8 a	1.77 a	7.20 a	9.54 a	18.1 c	89.5 e
		(+44)	(+44)	(+135)	(+65)	(+72)	(+66)	(+60)	(+78)	(+130)	(+122)	(+148)	(+60)	(+17)
	SA5(200)	43.0 e	47.9 f	12.5 de	1.19 f	0.292 g	1.48 f	0.260f	40.0 ab	1.46 c	6.34 b	7.82 c	16.5 d	86.1 f
		(+35)	(+37)	(+121)	(+49)	(+40)	(+46)	(+48)	(+70)	(+90)	(+43)	(+103)	(+46)	(+13)

SDW = Soot dry weight; RDW= Root dry mass; K^+/Na^+= K^+ to Na^+ ratios in shoot; Chl a = chlorophyll a; Chl b = chlorophyll b; Chl a+b=Total chlorophyll; Car = carotenoids; CAT = catalase; APX = ascorbate peroxidise; SOD = superoxide dismutase; Pn= photosynthetic rate; Ci= intercellular CO2 concentration; Gs= stomatal conductance; P= Proline; SA = foliar application Salicylic acid levels; Values within a group in a row bearing different superscripts are significantly different at P ≤ 0.05 .The numbers in brackets represent the percentage of changes.
